# Unraveling the role of amphisomes in mast cell secretory granule fusion and exosome release

**DOI:** 10.20517/evcna.2024.96

**Published:** 2025-03-24

**Authors:** Irene Tsilioni

**Affiliations:** Department of Immunology, Tufts University School of Medicine, Boston, MA 02111, USA.

**Keywords:** Mast cells, secretory granules, amphisomes, exosomes, fusion, mechanisms

## Abstract

Mast cells (MCs) play a crucial role in immune responses by storing and releasing inflammatory mediators from secretory granules (SGs). The biogenesis, maturation, and fusion of these granules with the plasma membrane regulate inflammation, immune cell recruitment, and tissue homeostasis. However, the exact mechanism underlying this process remains unclear. Recent studies have identified a novel mechanism of SG fusion involving amphisomes, hybrid organelles formed by the fusion of late endosomes and autophagosomes. This process not only facilitates SG enlargement but also promotes the release of exosomes, small vesicles crucial for intercellular communication and immune modulation. In particular, Omari *et al.* delve into the molecular machinery governing amphisome formation and SG fusion, focusing on key players such as Rab5, PTPN9, CD63, and phosphoinositides (PIs). They propose a dynamic model wherein amphisomes act as intermediates in SG maturation, promoting homotypic fusion events that regulate SG content and size. A critical aspect of this process is the lipid signaling cascade, particularly involving PI4K and CD63, which coordinates SG fusion and exosome release. These findings challenge the conventional view of SGs as static storage compartments, positioning them as dynamic hubs of vesicle trafficking and secretion. By elucidating the role of amphisomes and lipid signaling in SG biology, this study offers a significant shift in understanding and introduces new concepts that could drive future research. This commentary, while endorsing the authors’ key conclusions, also highlights important questions regarding the functional implications of these novel mechanisms and their potential therapeutic applications.

## MAIN TEXT

Regulated exocytosis is a vital process in eukaryotic cells, enabling communication and defense by releasing specialized cargo, such as hormones, enzymes, neurotransmitters, and inflammatory mediators, from secretory granules (SGs) in response to external signals^[1-3]^. Despite their critical role in endocrine, exocrine, neuronal, and immune cells, the mechanisms of SG biogenesis remain incompletely understood.

Mast cells (MCs) are key players in immune responses that use SGs to store and release inflammatory mediators such as histamine, proteases, cytokines, and chemokines^[4-7]^. Biogenesis, maturation, and eventual fusion of these granules with the plasma membrane for exocytosis are essential for regulating inflammation, immune cell recruitment, and tissue homeostasis, ensuring proper immune responses^[3]^. Dysregulated exocytosis can lead to pathological conditions like allergies and autoimmune diseases, highlighting the importance of understanding SG biogenesis in MCs for the development of potential therapeutic strategies.

### A novel model for SG fusion in MCs

One of the most significant contributions of the study by Omari *et al.* is the proposal that SG fusion in MCs is driven by the endocytic and autophagic systems working in concert^[8]^. Traditionally, SGs have been classified into two distinct types: those derived from the Golgi apparatus and those originating from the endocytic system^[9,10]^. However, as the authors point out, this dichotomy is increasingly outdated, especially in the case of MCs, where SGs exhibit both Golgi and lysosomal features. The fusion of Golgi-derived SGs with late endosomes and the subsequent formation of amphisomes, organelles that combine both endocytic and autophagic characteristics, provides a novel and dynamic model for SG maturation.

The concept of amphisomes as intermediates in SG fusion is an exciting and thought-provoking idea. The authors convincingly demonstrate that amphisomes, formed by the fusion of endosomes with autophagosomes, act as critical hubs for subsequent fusion events that result in SG enlargement^[8]^. This model not only challenges the simplistic view of SGs as static storage compartments but highlights their dynamic role in MC secretion, where their size and content can be tightly regulated. Furthermore, the involvement of the non-receptor tyrosine phosphatase PTPN9 in regulating amphisome formation and SG fusion presents a fascinating new avenue for investigating how phosphatases contribute to membrane trafficking and vesicle maturation.

Additionally, the study’s focus on the role of phosphoinositides, particularly PI4K and CD63, in regulating SG fusion provides an important molecular framework for understanding SG maturation. The authors propose that CD63 recruits PI4K to the SGs, promoting the synthesis of PI(4)P, a crucial lipid for the production of PI(3,4,5) P3, which activates PTPN9^[8]^. This cascade of lipid signaling ensures the proper coordination of SG fusion events and exosome release. By linking lipids to the regulation of SG function, the study sheds light on a previously underexplored aspect of SG biology: the role of membrane.

In particular, the authors’ suggestion that the interplay between PI4K and CD63 is essential for the regulated release of exosomes is groundbreaking^[8]^. Exosomes, which are small extracellular vesicles that play critical roles in cell communication and immune responses, have long been studied in other cell types, but the process of their release from MCs has been less well understood^[11]^. By connecting exosome release to the fusion of SGs with amphisomes, the authors offer a new perspective on how MCs can fine-tune their secretory responses based on external stimuli. This adds a layer of complexity to our understanding of MC function, suggesting that MCs may not simply release mediators in bulk, but can modulate both the quantity and type of mediators they secrete, including exosomes.

### Exploring unresolved issues and future perspectives in SG fusion

(a) Amphisomes: Canonical or Non-canonical? This study identifies amphisomes as intermediates in SG fusion, formed by endosome-autophagosome fusion regulated by PTPN9. Recent research, however, indicates that LC3 may conjugate to single membranes in processes such as LC3-associated endocytosis (LANDO) and LC3-dependent EV loading and secretion (LDELS)^[12]^. If amphisomes form via this non-canonical pathway, it could challenge traditional autophagosome biology and offer new insights into vesicle trafficking in MCs and other secretory cells.

(b) Functional Implications of SG Fusion: While SG fusion and exosome release are linked to immune response regulation, the functional implications remain unclear. Larger SGs may be involved in chronic or adaptive responses, while smaller SGs might be better suited for acute reactions. Furthermore, stimuli such as IgE/Ag and Substance P (SP) affect SG fusion patterns, raising questions about whether amphisome fusion influences exocytosis modes (e.g., full exocytosis, kiss-and-run), potentially affecting immune responses.

(c) Lipid Signaling in SG Fusion and Fission: The study emphasizes the role of phosphoinositides, like PI(3,4,5)P3 and PI(3)P, in SG fusion, but the involvement of other lipids in regulating SG size and release is still unclear. Lipid composition may serve as a switch for SG fusion, fission, or alternative exocytosis. Lipid remodeling could also influence SG populations and their secretory profiles.

(d) Therapeutic Implications: Targeting SG Fusion: The authors’ findings have significant therapeutic implications, particularly in immune modulation. Since SG fusion regulates the release of exosomes and soluble mediators from MCs, targeting molecules like PTPN9, CD63, or PI4K could offer new strategies for treating allergic diseases, chronic inflammation, or cancer. However, manipulating SG fusion could disrupt immune surveillance or worsen allergic reactions. These complexities highlight the need for further research to understand SG fusion before clinical application.

### Conclusion: a paradigm-shifting study

In conclusion, Omari *et al.* present a novel model for SG biogenesis and fusion in MCs, emphasizing the roles of amphisomes, phosphoinositides, and the endocytic/autophagic systems in SG maturation^[8]^. This challenges current paradigms and opens new research avenues. However, unresolved questions regarding amphisome formation and the functional implications of SG fusion highlight key areas for future investigation. This study delivers crucial insights that could advance our understanding of MC biology and drive novel therapeutic strategies for immune diseases, with a significant impact on the field.

## DECLARATIONS

### Copyright

© The Author(s) 2025.
